# A complex nursing intervention of complementary and alternative medicine (CAM) to increase quality of life in patients with breast and gynecologic cancer undergoing chemotherapy: study protocol for a partially randomized patient preference trial

**DOI:** 10.1186/s13063-014-0538-4

**Published:** 2015-02-15

**Authors:** Nadja Klafke, Cornelia Mahler, Cornelia von Hagens, Justine Rochon, Andreas Schneeweiss, Andreas Müller, Hans-Joachim Salize, Stefanie Joos

**Affiliations:** Department of General Practice and Health Services Research, University Hospital Heidelberg, Voßstr. 2, D-69115 Heidelberg, Germany; Department of Gynaecological Endocrinology and Reproductive Medicine, University Women’s Hospital Heidelberg, Im Neuenheimer Feld 440, D-69120 Heidelberg, Germany; Institute of Medical Biometry and Informatics, University of Heidelberg, Im Neuenheimer Feld 305, D-69120 Heidelberg, Germany; National Center for Tumor Diseases, University Hospital, Im Neuenheimer Feld 460, D-69120 Heidelberg, Germany; Community Hospital Karlsruhe, Moltkestraße 90, D-76133 Karlsruhe, Germany; Central Institute of Mental Health, Mental Health Services Research Group, Medical Faculty Mannheim/Heidelberg University, Mannheim, D-68159 Germany; Institute of General Practice, University Hospital Tuebingen, Oesterbergstr. 9, D-72074 Tuebingen, Germany

**Keywords:** Breast and gynecologic malignancies, Integrative oncology, Complementary and alternative medicine (CAM), Counseling and nursing, Complex interventions, Health services research, Quality of life

## Abstract

**Background:**

Health-related quality of life (HRQoL) is most adversely affected in cancer patients between diagnosis and the end of chemotherapy. The aim of the Complementary Nursing in Gynecologic Oncology (CONGO) study is to assess the effectiveness of a complex nursing care intervention of CAM to increase HRQoL in cancer patients undergoing chemotherapy.

**Methods/design:**

CONGO is a prospective partially randomized patient preference (PRPP) trial including adult women diagnosed with breast and gynecologic cancer starting a new chemotherapy regimen. Patients without strong preferences for CAM will be randomized to usual nursing care or complex nursing care; those patients with strong preferences will be allowed their choice. The intervention consists of three interacting and intertwined elements: CAM nursing intervention packet, counseling on CAM using a resource-oriented approach and evidence-based informational material on CAM.

Primary outcome data on participants’ HRQoL will be collected from baseline until the end of treatment and long-term follow-up using the EORTC-QLQ-C30. Secondary outcomes include nausea, fatigue, pain, anxiety/depression, social support, self-efficacy, patient competence, spiritual wellbeing, and satisfaction with care. Accompanying research on economic outcomes as well as a mixed-methods process evaluation will be conducted.

A total of 590 patients (236 patients in the randomized part of the study and 354 patients in the observational part of the study) will be recruited in the two outpatient clinics. The first analysis step will be the intention-to-treat (ITT) analysis of the randomized part of the trial. A linear mixed model will be used to compare the continuous primary endpoint between the intervention and control arm of the randomized group. The observational part of the trial will be analyzed descriptively. External validity will be assessed by comparing randomized with nonrandomized patients.

**Discussion:**

Cancer patients are increasingly using CAM as supportive cancer care, however, a patient-centered model of care that includes CAM for the patient during chemotherapy still needs to be evaluated. This protocol has been designed to test if the effects of the intervention go beyond potential benefits in quality-of-life outcomes.

**Trial registration:**

German Clinical Trials Register (DRKS), DRKS00006056 (15 April 2014).

## Background

Gynecologic malignancies, including breast cancer, constitute approximately 95,600 new cases per year, which is nearly half (43%) of all malignancies in women in Germany [1]. It is known that health-related quality of life (HRQoL) is most negatively affected from the time of diagnosis through the completion of chemotherapy [2]. In this demanding phase, many patients are looking for help and orient themselves towards the field of complementary and alternative medicine (CAM). Consequently, the use of CAM by cancer patients has increased considerably over recent years [3], particularly in breast cancer populations [4].

Research suggests that cancer patients use CAM alongside their conventional therapy for a variety of reasons [5,6]. For example, patients use CAM to improve their HRQoL (for example, to relieve stress and anxiety and to alleviate side-effects of the chemotherapy) or they are motivated by the desire to maximize the success of treatment through their own actions (‘self-efficacy’) [3,7]. Overall, CAM use constitutes a form of coping strategy that helps cancer patients and their involved significant others (SOs), like family members and close friends, to overcome the distressing cancer diagnosis [8].

In a German sample of 1,030 patients with gynecologic and breast cancer, CAM was used by 49% of all women (50% in breast cancer patients versus 44% in gynecologic cancer patients). In this study, CAM users less frequently stated an overall deterioration of their health status (35%) compared to nonusers (50%) [9]. These numbers are in line with international studies where the rate of CAM use in women with breast cancer has been reported to be even higher (up to 75%), with the highest percentages in women undergoing chemotherapy [10].

Worldwide, oncologists have acknowledged that CAM may contribute positively to patients’ psychological and physical well-being and have started to develop guidelines on integrating CAM within a quality-controlled, evidence-based strategy [11-13] concluding that there is a lack of expert guidance available to patients. It has been suggested that oncology nurses are in a key position to evaluate which symptoms or side-effects patients are experiencing and to identify which CAM therapies may be helpful for symptom control related to treatment and psychological distress due to their cancer and the chemotherapy [14,15].

Internationally, oncology nurses have responded to this high patient demand for CAM by incorporating selected CAM therapies such as acupressure or the application of essential oils by massage, packs or compresses within their everyday practice. In several countries (for example, the United States, Canada, and the United Kingdom.), nursing schools have resumed the incorporation of CAM into their educational curricula and nursing standards [16]. Furthermore, nurse-led CAM education programs for patients or services for individualized patient consultations have been established [17].

In Germany, CAM therapies have been increasingly integrated into nursing practice, particularly in oncology and palliative care settings [18-20]. These kinds of interventions, often accompanied by individual patient counseling, require additional time inputs to daily routine care. Therefore, staffing shortages and time restrictions can impede the integration of CAM into nursing care. Attempts to do so, with the existing conditions in our health care system, have not been frequent, and the effectiveness of CAM nursing interventions in routine clinical practice still remains to be evaluated. Considering both the demand of cancer patients for CAM and also the possible risks in certain cases due to poor information and/or communication [21], CAM interventions should be systematically researched and, based on evidence, structures accordingly adapted.

Levine’s conservation model [22] provides a valuable theoretical framework for guiding and promoting a complex CAM intervention aimed at increasing health-related quality of life in cancer patients undergoing chemotherapy. According to this model, the individual is posited as a spiritual, holistic being experiencing meaning in social relations [22]. For maintaining or re-gaining health, and experiencing a state of wholeness, the individual tends to adapt by the conservation of energy, structural integrity, personal integrity, and social integrity. Based on Levine’s conceptual model, it has been suggested that nurses can support cancer patients in the process of adaptation after a distressing cancer diagnosis and treatment by addressing the patients’ conservation strategies, so that patients can find back to a state of integrated and balanced wholeness [23,24]. These conservation principles guided the development of the current study’s intervention, and the identification of patient-centered outcomes.

Thus, the Complementary Nursing in Gynecologic Oncology (CONGO) study was initiated as a prospective partially randomized controlled intervention study to investigate the effects of a complex nursing care intervention on HRQoL and a wide range of clustered symptoms in female cancer patients undergoing chemotherapy. As patients are rarely neutral towards CAM, this study considers patients’ preferences for receiving or not receiving the intervention. By assessing the quality of life outcomes and evaluating the processes of the intervention, it is anticipated that the CONGO study will provide evidence-based information on integrated health care services relevant for the treatment of cancer.

## Methods/design

The study protocol received the approval by the ethics committee of the University of Heidelberg (S-008/2014) as well as the ethics committee of the State Medical Council of Baden-Wuerttemberg (B-F-2014-037), and is registered with German Clinical Trials Register (https://drks-neu.uniklinik-freiburg.de/drks_web/) under DRKS00006056.

### Objectives

The primary outcome of the CONGO study is cancer patients’ HRQoL. It will be assessed if cancer patients, starting a new chemotherapy regime, benefit from a complex nursing care intervention, consisting of a CAM intervention packet (for example, aromatherapy, massage, compress, acupressure), counseling on self-help CAMs, and evidence-based informational material on CAM, regarding HRQoL in comparison to patients receiving usual nursing care.

Secondary outcomes are to assess the effects of the complex intervention on a wide range of physical and psychosocial symptoms including nausea, fatigue, pain, anxiety/depression, social support, self-efficacy, patient competence, spiritual wellbeing, patient satisfaction, and the quality of life of patients’ significant others (SOs). Furthermore, accompanying research on health economic outcomes will be conducted.

In addition, the acceptability and feasibility of the complex intervention will be evaluated in the two recruiting centers by providing a thorough process evaluation and information about the provision of integrated cancer care in outpatient cancer services. Therefore, quantitative data will be collected that will be supplemented with qualitative interview data involving healthcare staff, patients, and their SOs.

### Design of the study

The CONGO study has been designed as a partially randomized patient preference (PRPP) trial for the following reasons: randomized clinical trials (RCT) are the gold standard for establishing the efficacy of medical treatments [25,26]. Randomization has the key advantage of controlling for both known and unknown confounders and the elimination of their influence on the treatment outcome. However, patients often have a preference for a treatment. In such case, randomization might fail (preference effect) [27]. Patients with strong preferences are more likely to decline participation in a RCT because randomization does not guarantee that they will get what they want. If a large number of patients refuse randomization, study results may not be representative of the whole population, and the external validity of the trial will be negatively affected. On the other hand, if patients with strong preferences consent to randomization, this may affect the internal validity of the trial because randomization cannot deal with the post-randomization effects of patients’ preferences on outcomes [28].

**Figure 1:**
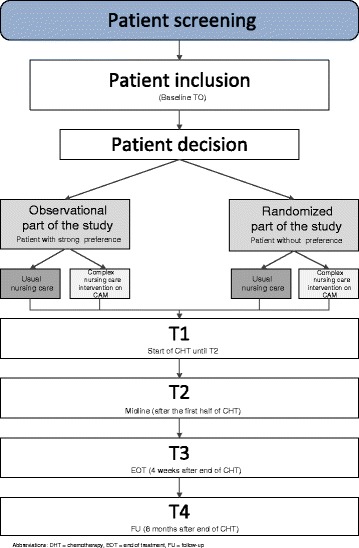
**Study flow chart.** CHT, chemotherapy; EOT, end of treatment; FU, follow-up.

If the current study would follow a purely standard RCT design, patients with strong preference for CAM will be pleased when assigned to the ‘complex nursing care intervention on CAM’ group and disappointed when assigned to the ‘usual nursing care’ group. The preferences will, thus, affect the HRQoL assessment within the trial. Therefore, the current study has been designed as a partially randomized patient preference (PRPP) trial. Patients with strong preferences will be allowed their choice, and those without (strong) preferences will be randomized to usual nursing care or complex nursing care (see Figure 1).

The PRPP design allows for recruiting a higher number of patients, which increases the external validity and generalizability of the results [29]. Measurement bias due to patients being disappointed by their treatment allocation will be minimized, as these patients will not be randomized and allowed their desired treatment without randomization. In addition, the PRPP design enables comparisons between patients with and without a preference and an exploration of patients’ characteristics associated with preference.

A disadvantage of the PRPP design is that the preference (= observational) arms suffer from selection bias and are therefore susceptible to unmeasured confounding. Primary analysis is therefore usually being performed on the two randomized treatment arms, which should be large enough to reliably answer the study question.

### Participants and setting

To represent everyday conditions, and for obtaining a high external validity and generalizability, female cancer patients will be recruited under broad inclusion criteria (for example, all adult women diagnosed with breast or gynecologic malignancies starting a new chemotherapy regimen) from different levels of care (university hospital and community hospital). All patients registering for a new adjuvant or neoadjuvant chemotherapy regimen will be informed about and invited for study participation, irrespective of their participation in other studies. The only exclusion criteria are insufficient knowledge of the German language, cognitive impairment, and inability to give informed consent.

### Recruitment and randomization

Informed consent will be obtained from all women before study entry. Breast and gynecological cancer patients registering for a new chemotherapy regime at the National Centre for Tumour Diseases (NCT) Heidelberg and the Community Hospital Karlsruhe will be informed by flyer, their treating oncologist, or nursing staff about the CONGO study. Then, a CAM-trained nurse will approach the potential participant, briefly inform her about the study and ask her if she would like to have further information. Those agreeing will be provided with details of the study verbally and in written form via the study information leaflet. Patients who agree to participate will be asked to sign the consent form. After informed consent, baseline assessments will be conducted. The nurse will assert if the potential participant has a strong preference to receive the CAM nursing intervention and allocate her into the observational part of the study if applicable. All other participants will be allocated into the randomized part.

The CAM-trained nurses will randomize the study participants using *randomizer.at*, which is an online central randomization service. Participants will be stratified by cancer center (NCT Heidelberg/Community Hospital Karlsruhe) and stage of cancer (curative treatment/palliative treatment), and then randomly allocated at a ratio of 1:1 to the intervention or control arm of the randomized part of the trial. Blocked randomization with varying block sizes will assure that the numbers in the arms are balanced, while the nurses are not being given the possibility to deduce the next allocation. Further, allocation concealment will be ensured, as the randomization service does not release the code for the randomization done by the CAM-trained nurse administering the intervention. Thus, it will be assured that the recruiter has no knowledge about the random sequence and hence cannot influence participants due to possible bias.

### Intervention group

The complex intervention [30,31] consists of three interacting and intertwined elements tailored to the patients’ needs: CAM nursing intervention packet, counseling on CAM using a resource-oriented approach and evidence-based informational material on CAM.

This complex intervention combines existing evidence and practical experience and was developed by an expert team consisting of a qualified nursing instructor, nursing staff experienced with CAM in cancer patients, study nurses, medical doctors, and researchers specialized in psycho-oncology, complementary therapies, and health services research. The CAM nursing intervention packet was composed during a two-day workshop in February 2014. It was then finalized after several meetings and iterative discussions, modifying and concisely summarizing the intervention packet to meet the study’s aim. The final intervention packet targets a range of physical and psychological symptoms affecting cancer patients’ HRQoL during chemotherapy administered in an outpatient setting. The CAM-trained nurse will select an intervention from the nursing intervention packet and tailor it to the patient’s individual needs. In addition, patients receive standardized CAM information sheets with instructions on how to conduct selected CAM practices at home. The selection of these CAM information sheets will depend on the patient’s physical and psychological concerns, as well as on preferences, and the nurse’s recommendation. The information sheets will be included in a diary patients have to keep during the study.

When patients visit the cancer center for their first cycle of chemotherapy, they will receive an initial counseling interview. In this interview the nurse will explain the potential side-effects of the chemotherapy and how to prevent or alleviate them with usual nursing care or the complex nursing care intervention on CAM. During this interview, the nurse will also provide resource-oriented counseling, in particular informing the patient about how she can activate her own inner resources (for example, following a balanced diet and exercise plan, keeping a positive mind-set by meeting regularly with their family and friends, getting on with or taking up a new hobby). This counseling is based on concepts of patient competence and empowerment [32-34], considering patients’ request to actively do more to complement their conventional treatment. CAM-trained oncology nursing staff will also be responsible for individual patient counseling on CAM, guiding the patient on how to cope with physical and psychological symptoms at home. These nurses will be a liaison person on CAM therapies whom the patient can contact anytime while on the chemotherapy regime. Furthermore, study participants will receive an evidence-based information booklet and a DVD including further answers and guidance about possibilities for complementing their conventional cancer treatment with CAMs. In providing patients further evidence-based CAM material, patients’ self-management of their disease will be promoted in a safe way. The development process of the complex intervention and the intervention packet will be described in detail in a separate publication.

### Control group

Control group participants of both the randomized and observational part of the study will receive usual care according to the clinic’s standard. All patients receive medical treatment and supportive care including the administration of analgesics or antiemetics. Other supportive care procedures including counseling or inserting (CAM) products or techniques that are not yet standardized in the two recruiting centers but that will be documented during the whole study phase. Control group patients will participate in all data collection assessments (questionnaires and patient diary) but will not receive any CONGO-related interventions mentioned above.

### Training of nursing staff

The intervention in the current study will be conducted by nursing staff, who attended two training modules composed for the study in order to achieve standardization between the two centers. The first module consisted of training on CAM therapies for oncology nursing and was taught by a qualified CAM nursing instructor (www.gisela-blaser.de). All CAM therapies, which will be applied in the current study, were performed in the session. Additionally, a CAM nursing manual including supplementary information for the application of these CAM therapies was provided. In the second module, the nurses received training on specific communication/counseling within the intervention. In a simulation exercise with lay persons (students of the University Heidelberg), the nurses were trained to conduct study briefings and resource-oriented counseling interviews (see Intervention group for further information), and they received manuals for the study briefing and patient counseling.

### Outcome measures

#### Schedule

**Figure 2:**
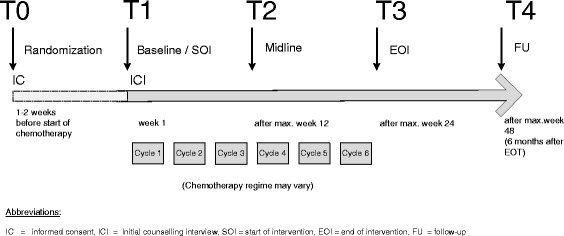
**Timeline.** EOI, end of intervention; FU, follow- up; IC, informed consent; ICI, initial counseling interview; SOI, start of intervention.

All outcome measures are summarized in Table 1. There are five specific assessment time points, which are illustrated in the timeline (see Figure 2):

**Table 1:** **Assessments and instruments used in the Complementary Nursing in Gynecologic Oncology (CONGO) study**

**Outcomes**	**Instrument**	**T0**	**T1 Baseline/SOI**	**PD Every week**	**PD Once per cycle**	**T2 Midline**	**T3 EOI**	**T4 FU**
**Primary outcome**								
Health-related quality of life	EORTC-QLQ-C30		X	X		X	X	X
**Secondary outcomes**								
Nausea	MAT				X			
Fatigue	FACIT-F		X			X	X	X
Pain (general)	VAS		X			X	X	X
Anxiety, depression	PHQ-9		X			X	X	X
Social support	F-SozU K-14		X				X	X
Self-efficacy	SES6G		X			X	X	X
Patient competence	FEPK 2-57		X				X	X
Spiritual wellbeing	Sp-REUK-15		X				X	X
Patient satisfaction	EORTC-QLQ-PATSAT32						X	
Accompanying research (health economy)	Mannheimer Modul RV		X			X	X	
Quality of life SOs	WHOQOL-BREF		X				X	X
	CQOLC		X				X	X
**Others** Socio-demographic factors		X						
Cancer characteristics		X						
Medical history		X						
Treatment data			X		X	X	X	X

*EOI* end of intervention, *FU* follow-up, *PD* patient diary, *SOI* start of intervention, *SO* significant other.

- T0: Baseline assessment 1 to 2 weeks before the start of chemotherapy).
- T1: Start of intervention (SOI).
- T2: Mid-line (after the first half of the chemotherapy regimen; maximum of 12 weeks).
- T3: End of intervention (EOI) (after the end of the chemotherapy; maximum 24 weeks).
- T4: Follow-up, 6 months after the completion of chemotherapy (after maximum of 48 weeks).

### Instruments

All study participants, from both the randomized and observational part of the study will be asked to answer the following instruments in paper-based form (questionnaire pack and patient diary).

### Quality of life (patients)

The main outcome measure HRQoL will be measured with the global quality of life scale of the European Organization for Research and Treatment of Cancer Quality of Life Questionnaire (EORTC-QLQ-C30) [35-37]. The QLQ-C30 is a 30-item questionnaire developed to assess the HRQoL and five multi-item functional scales (physical, role, emotional, cognitive, and social function), three multi-item symptom scales (fatigue, pain, and nausea/vomiting), and six single items assessing further physical symptoms (appetite loss, constipation, diarrhea, dyspnea, and insomnia) and financial difficulties. The global quality of life will be calculated from two items (item 29 ‘overall health last week’ and item 30 ‘overall HRQoL last week’) , which are measured in seven interval Likert scales and then combined and transformed into a scale between 0 and 100 points. In the planned trial, the HRQoL will be assessed at all assessment points T1, T2, T3, T4, and weekly in the patient diary.

### Nausea

Nausea will be assessed with the Multinational Association of Supportive Care in Cancer Antiemesis Tool (MAT), which is an eight-item scale assessing both acute and delayed nausea [38]. The MAT is designed to be used once per cycle on two days, and convinces for employment in the current study with regard to practicability and user-friendliness. The MAT will be included in the patient diary.

### Fatigue

The Functional Assessment of Cancer Therapy - Fatigue (FACT-F) subscale contains 13 fatigue-specific items, which patients can rate on a 5-point Likert scale; high internal consistency and construct validity have been reported [39-41]. The German version of the FACIT did undergo a specific linguistic validation process (FACIT translation methodology), and is thus considered as appropriate for use in the current study [42].

### Pain

The global rating of pain is assessed by a single-item pain visual analog scale, which measures patient’s severity of pain over the previous week [43].

### Anxiety, depression

Anxious and depressive symptoms are measured with the nine-item Patient-Health-Questionnaire (PHQ-9), which is a validated tool for measuring DSM-IV depressive disorders [44]. The PHQ-9 is a shorter and more user-friendly form than other depression measurements and thus is being employed in the current study.

### Social support

The original questionnaire about social support (Fragebogen zur Sozialen Unterstützung, F-SozU) is a 54-item questionnaire from Germany covering a range of items about patients’ perception of emotional support, practical support, social integration, burden from the social network, reciprocity, availability of a confident, and satisfaction with social support; and has provided high reliability and validity [45]. We will employ the short F-SozU-14, as we anticipate that a reduction of items will contribute to patients’ compliance in answering the whole questionnaire pack, and reduce potential burden arising from high time exposure.

### Self-efficacy

Patients’ self-efficacy will be measured with the German version of the Self-Efficacy for Managing Chronic Disease 6-Item Scale (SES6G). This assessment tool has provided high internal consistency and good convergent construct validity [46]. Patients will be asked on 10-point Likert scales to indicate how confident they perceive themselves in handling their chronic illness.

### Patient competence

The concept of patient competence will be measured with the FEPK 2–57, which is a 57-item questionnaire involving five problem-focused and three emotion-focused subscales [34]. Patients will be asked on 5-point Likert scales how they perceive their competencies in coping with their disease with regard to employing self-managing strategies and communication with others. The questionnaire has provided satisfactory reliability; while external validity still needs to be evaluated and a short version composed.

### Spiritual wellbeing

Cancer patients’ quality of life [47] and CAM use [48] are associated with their spiritual well-being, which we will measure with the ‘Spiritual and Religious Attitudes in Dealing with Illness’ (SpREUK) instrument [49,50]. On 5-point Likert scales, participants are asked the extent to which they experienced aspects of search, faith, and reflection in the past week; thereby assessing how their confrontation with an existential illness resulted in their essential need to find meaningful answers and practices. We will employ the short form SpREUK-15 , which has provided high internal consistency and is considered to be suited for oncology populations [51].

### Patient satisfaction

For evaluating patients’ perceptions on the quality of medical and nursing care, we will employ the EORTC-QLQ-PATSAT32. This questionnaire has been validated in a cross-cultural context and provides excellent values for reliability and convergent validity [52].

### Quality of life (patients’ significant other)

The WHOQOL-BREF is a validated assessment tool for measuring generic quality of life in individuals [53]. We will employ this internationally widely employed tool to assess if patients’ HRQoL correlates with the QoL of their significant others (SOs). In addition, patients’ SOs will be asked to answer the German version of the Caregiver Quality of Life Index-Cancer (CQOLC), an instrument measuring specific and more accurate areas of the quality of life of significant others of patients with cancer [54]. The German translation is accessible in the Patient-Reported Outcome and Quality of Life Instruments Database (PROQOLID), but the psychometric properties of this version have not yet been reported [55].

### Accompanying research on economic data

Kligler and colleagues have shown that an integrative medicine approach including holistic nursing and a healing environment in an inpatient oncology setting can decrease anxiety, fatigue, and the use of medications resulting in substantial cost savings in the care of oncology patients [56]. Such evidence provides the rationale for including health economic analyses into our study plan. Secondary health economy-related endpoints will include the following:

1. the cost of study treatments in intervention and control groups,
2. the total cost of health care utilization and productivity losses in intervention and control groups during follow-up,
3. the cost-effectiveness of trial intervention,
4. the quality adjusted life years (QALY) gain or loss in intervention and control groups during follow-up, and
5. the cost per QALY in intervention and control groups.

The health economy analyses include the following steps:

1. the assessment of the cost of the trial related intervention in the experimental groups and costs in the control groups,
2. the collection of health service utilization data in the control and intervention groups during the follow-up,
3. the assessment of longitudinal HRQoL data for transformation into preference measures and calculation of QALYs lost or gained during the follow-up, in order to calculate costs per QALY of interventions and controls, and
4. the calculation of the incremental cost-effectiveness ratio (ICER), cost-effectiveness acceptability curves (CEAC) and willingness-to-pay levels.

For collecting data on health services utilization during the follow-up, a scale [57] applied in international research will be adapted to the specific conditions of the German health care system and applied in the study sample (Mannheimer Module RV). To calculate QALYs, preferences generated from the EORCT-QLQ-C30 data will be used for patients [58], and WHOQOL-BREF data for patients’ SOs [53,59].

### Side-effects of chemotherapy

Adverse events of the conventional cancer treatment will be documented according to the international standard of the National Cancer Institute (NCI) Common Terminology Criteria for Adverse Events [60].

### Sample size and power considerations

Sample size was calculated for the global quality of life (Items 29 and 30 of EORTC-QLQ-C30) and was based on a two-group comparison using Student’s t-test because no reliable estimate of the correlation between repeated measures was available. Following the recommendation by Cocks *et al*. [36], a difference of 10 points between the randomized groups was considered clinically important (that is, ‘smallest medium difference’). This difference corresponds to a standardized effect of d = 0.4 according to Cohen. To detect d = 0.4 with a power of 1 - β = 80% using a two-sample t-test at a two-sided significance level α = 5%, a total of 200 patients in the randomized arms (100 per arm) will be required. The sample size calculation was performed using nQuery Advisor V7.0. We expect the power of the main statistical analysis to be higher than 80% because repeated measurements will be taken into account and the analysis will be additionally adjusted for relevant covariates such as HRQoL at baseline.

We assume that 40% of the patients have no or no strong preference and thus will be randomized. About 60% of the patients are assumed to have a strong preference for the CAM intervention. Therefore, we need to include 200 patients in the randomized arms (100 per arm) and we expect to include about 300 patients in the observational arms. Assuming up to 15% of patients may not have primary outcome data available, the sample size was adjusted to a total of 236 patients in the randomized arms (118 per arm) and about 354 patients in the observational arms.

### Data analysis

According to Schmoor *et al.* [61], the first analysis step of PRPP trials is the classical analysis of the randomized patients. These are the basic results of the trial and these results have internal validity. The primary objective of the present study is to determine the effectiveness of a complex nursing care intervention on CAM in women with breast and gynecologic cancer undergoing chemotherapy, and to show that the complex intervention results in higher HRQoL, compared to usual nursing care alone. The primary endpoint is the global HRQoL subscale of the EORTC QLQ-C30 questionnaire and is assumed to follow an approximate normal distribution [35].

A linear mixed model will be used to compare HRQoL under chemotherapy between the two randomized groups from treatment start to long-term follow-up (T1 to T4). This model takes into account the hierarchical structure of the data with repeated measurements nested in patients (that is, patients will be included as random effects in the intercept). Fixed effects will include treatment (usual nursing care versus complex nursing care intervention on CAM), the time of measurement (T1 to T4), the interaction of treatment and time, as well as the HRQoL baseline scores at T1, and the stratification variables stage of cancer (non-metastatic versus metastatic) and study centre (university hospital versus community hospital). Because the measurements are not equidistant in time, an unstructured covariance matrix will be estimated. For the main effect of treatment, the null hypothesis of no difference in group averages will be tested at the two-sided significance level of α = 0.05. Differences between short-term and long-term effects will be assessed by the interaction of treatment and time. The primary analysis will be performed for all randomized patients according to the intention-to-treat principle. As sensitivity analysis, the analysis will be repeated in the per protocol set, excluding patients with major protocol violations. Because longitudinal mixed models make use of the missing at random assumption, no imputation will be used to handle missing data in the primary analysis. However, sensitivity analyses will be conducted to assess the impact of missing data (for example, omitted measurements at some time point) by various approaches, including for example the ICA-r method (imputation according to reasons for missingness [62]), the last observation carried forward, and multiple imputation. Model fit will be assessed by visual inspection of residual plots. Descriptive analyses of secondary endpoints will be performed. All estimates will be presented with 95% confidence intervals. External validity will be assessed by comparing the randomized with the non-randomized patients. The comparison of the observational group will thereby facilitate the applicability of the results. The central part of the second analysis step is, thus, to assess whether treatment effects observed in the randomized patients are comparable with those observed in the two observational arms. If there are measurable differences between these two groups with regard to prognostic factors, the effect of these differences will be examined using propensity score methods. Propensity scores will be determined that reflect the patients’ preferences.

### Quality assurance

The study is planned in accordance with the German data protection law and ICH/GCP guidelines. External monitoring and consultations with an advisory board will ensure patient safety and highest possible data quality. Monitoring visits will be conducted on a regular basis by independent qualified study nurses of the Department of General Practice and Health Services Research, University Hospital Heidelberg. This monitoring will include pre-study-visits and monitoring visits using standard operating procedures with monitoring of complete eCRFs of at least 10% of the patients included and of core and safety data of the remaining patients. Furthermore, study nurses will check the provision of the interventions in both study sites on a random basis.

All instruments will be piloted to assure readability and limit missing item response. Survey instruments will be provided by the coordinating centre (Department of General Practice and Health Services Research) to minimize printing error or layout variation. The coordinating centre will be responsible for scanning of all questionnaires from both study sites, for query management and data consistency check. This study is supported by an external advisory board including experts/representatives of CAM, clinical pharmacology, oncology, study methodology, nursing science, statistical science and the patient perspective.

## Discussion

The CONGO study is the first prospective partially randomized patient preference trial investigating the impact of a nurse-led intervention involving both clinic and home-based CAM use in breast and gynecologic cancer patients undergoing chemotherapy in two outpatient cancer services. The findings from this study will add to the growing body of evidence of the effectiveness of CAM interventions for individuals with cancer, by testing several aspects relevant for the implementation of patient-centered models of integrative healthcare: (1) patients will receive cancer care including CAMs and individual counseling on CAM by oncology nurses; (2) patients’ preference to receive the CAM intervention will be considered; (3) patients’ empowerment resources will be activated in the initial and following individualized counseling interviews, by receiving standardized CAM instructions and an additional information booklet including evidence-based CAM for home use; (4) The effect of the intervention will be measured on HRQoL and a range of other physical and psychosocial symptoms; (5) Sustainability and long-term effects of this intervention will be assessed by follow-up; (6) A thorough process evaluation will provide explanation on succeeding or impedimental factors impacting concomitantly on the process of the intervention.

The majority of breast and gynecologic cancer patients receive adjuvant or neoadjuvant chemotherapy, which may help to cure their cancer, or prolong life, but also affects their HRQoL due to treatment side-effects or psychosocial concerns [63]. Research consistently demonstrates that cancer patients have a high interest in complementing their conventional cancer treatment by using CAMs [3,5], and some integrative cancer centers have incorporated patients’ preference in providing counseling and methods reflecting a holistic treatment approach [18]. There is, however, a lack of research investigating the effects of such integrated services on a range of patient-centered outcomes in cancer patients treated with chemotherapy [64]. Previous randomized trials mostly investigated the efficacy of one CAM therapy on primary and secondary outcomes [65-68]. Likewise, other effectiveness studies, including nonpharmacologic interventions focused on investigating cognitive or educational programs, however, excluded CAM therapies [69,70] not knowing if patients applied CAM nevertheless. The current study aims to address this aspect by supplying patients in the control group with a patient diary to collect data related to physical and psychological symptoms as well as other supportive therapies patients may apply. While many intervention studies with cancer patients aimed to target cancer-related fatigue (CRF) [71-73], the CONGO study is designed to investigate if cancer patients’ and their significant others’ quality of life will be affected by the complex CAM nursing intervention.

In contrast to efficacy studies, the current effectiveness study will consider several elements when analyzing participants’ HRQoL. The intervention targets common symptoms of patients undergoing chemotherapy. To address these symptoms, a range of CAM interventions will be administered to the patient in the outpatient centre at each cycle of chemotherapy. Some of these CAM interventions are already part of the clinics’ nursing standards [20]; however, they have not yet been standardized and evaluated. In the current study, the patients will also receive counseling on how to apply some of these CAM interventions at home between cycles. Plus, patients will receive an evidence-based information booklet on different CAM interventions as well as contact sources on other external CAM providers in case they prefer to pursue the wide CAM spectrum further on. As a chemotherapy regimen lasts very long (for example, 24 weeks) the patient will visit the outpatient clinic every 3 to 4 weeks that is, 6 to 8 times. It is likely that the patient builds a relationship with the assigned CAM nurse caring for her, which might also add to improvements in HRQoL. In the randomized group, we can account for potential confounders, and analyze the primary and secondary outcomes, but we cannot explain which single element of the interacting intervention agents has the highest impact on changes in HRQoL.

The shift from inpatient to outpatient cancer treatment has increased awareness of patients’ self-care strategies and also of their significant others (SOs) including family members and close friends [74,75]. Cancer has become a family disease affecting not only the individual with the cancer diagnosis, but also involving their SOs who often are experiencing high levels of distress [76] while accompanying and supporting their loved one going through the treatment stages and adjusting to a new life situation [77]. A strength of the current study is the consideration of patients’ SO, whose QoL will be assessed T1, T3, and T4; and who will be interviewed during the process evaluation to gain a better understanding about their perceptions on the cancer patient’s experiences with the complex nursing care intervention on CAM. The interventions have been primarily composed for the patient receiving chemotherapy, and therefore exclude couple-based methods; however, some of these home-based interventions can be administered by the SOs (for example, preparing the tea, conducting the massage). Thus, the current study will provide further information in highlighting if the shared CAM use contributes to bonding and improvements of QoL in cancer patients’ SOs [74,75].

A further strength of the CONGO study is the interprofessional aspect, which takes into account the collaboration between health professionals in the design of the study as well as in the composition of the complex nurse-led intervention. To our knowledge, this is one of the first nurse-led CAM intervention studies in Germany that can assess nurses’ relevant contribution in promoting HRQoL during cancer therapy.

Another unique aspect of the CONGO study is patients’ choice of the study arm. Due to high interest and prevalence of CAM in cancer populations, we expect that 60% of participants will decide for the intervention and will therefore be allocated into the observational study arm. This study arm will be analyzed descriptively and will be used to assess the external validity of the study results. However, it will be a challenge to recruit enough participants for the randomized arm.

The logistic implementation of the CONGO study presents challenges in recruiting the two centers and the healthcare staff involved. Several meetings, involving mutual exchange visits of the involved nursing staff have taken place to facilitate the planning and coordination of the procedure of the study, and to find solutions for recruiting enough patients for the randomized arm. During these pre-study meetings, how the study participants can be recruited without any interruptions for the outpatient clinic routine has been elaborated, as well as how to care for and treat the study participants of the different study arms - with and without receiving the intervention. The organization of the study requires a flexible schedule of the CAM-trained nurses, as they have to inform the patients about the study, collect their informed consent, conduct the initial counseling and follow-up interviews, as well as conduct the intervention while the patient receives chemotherapy. In addition, it will be expected that the CAM-trained nurse cares about the data collection and documents the implementation of the intervention in an electronic case report file (eCRF).

In summary, the CONGO study has been designed to evaluate the effectiveness of an intervention involving CAM therapies and counseling on CAM as complementing the supportive care of breast and gynecologic cancer patients undergoing chemotherapy. It is hypothesized that this intervention increases HRQoL and clustered symptoms over the chemotherapy regimen and follow-up in this outpatient population, and that this is highly relevant to know in the context of promoting and advancing integrative cancer care/clinics.

## Trial status

The trial has received funding in December 2013 and ethical clearance in Mai 2014. At the time of submission of this protocol, the intervention has been standardized and nursing staff has been trained. Recruitment began in July 2014. We anticipate that all data will be collected by May 2016.

## Abbreviations

CHT: Chemotherapy
eCRF: Electronic case report file
EOT: End of treatment
EOI: End of intervention
FU: Follow-up
IC: Informed consent
ICI: Initial counseling interview
PD: Patient diary
SOI: Start of intervention
SOT: Start of treatment
SO: Significant other
